# *Notes from the Field:* Severe Medetomidine Withdrawal Syndrome in Patients Using Illegally Manufactured Opioids — Pittsburgh, Pennsylvania, October 2024–March 2025

**DOI:** 10.15585/mmwr.mm7415a3

**Published:** 2025-05-01

**Authors:** Simon J. Ostrowski, Kenichi Tamama, William J. Trautman, Darien L. Stratton, Michael J. Lynch

**Affiliations:** ^1^Division of Medical Toxicology, University of Pittsburgh School of Medicine, Pittsburgh, Pennsylvania; ^2^University of Pittsburgh Medical Center, Pittsburgh, Pennsylvania;^ 3^Department of Pathology, University of Pittsburgh School of Medicine, Pittsburgh, Pennsylvania.

**Summary**

**What is already known about this topic?**

Medetomidine is an increasingly common adulterant of illegally manufactured opioids.

**What is added by this report?**

During October 2024–March 2025, a total of 23 adult patients who used illegally manufactured opioids sought treatment within a health care system in Pittsburgh, Pennsylvania. All exhibited severe autonomic hyperactivity, and most required dexmedetomidine infusion and intensive care unit–level management. Medetomidine metabolites were detected in all 10 patients for whom retrospective analysis was performed, despite only two having detectable parent compound (medetomidine) on comprehensive urine drug screening.

**What are the implications for public health practice?**

Health care providers in regions where medetomidine has been detected in the drug supply should be prepared to manage a severe withdrawal syndrome among patients who use illegally manufactured opioids, even if drug testing for medetomidine is negative.

Various adulterants within the illegally manufactured opioid supply have emerged in the United States during the last several years (*1*–*3*). Medetomidine, an alpha-2 adrenoreceptor agonist that is not approved for human use in the United States, became increasingly detected in the drug supply in 2024. Medetomidine is a racemic mixture of levomedetomidine and dexmedetomidine, an intravenous sedative used in the critical care setting. A clinical syndrome of autonomic hyperactivity (i.e., hypertension, tachycardia, and agitation) has been observed after abrupt discontinuation of dexmedetomidine (*4*).

## Investigation and Outcomes

During October 2024–March 2025, the University of Pittsburgh medical toxicology service evaluated 23 patients at two Pittsburgh, Pennsylvania hospitals who experienced severe autonomic hyperactivity after abrupt cessation of illegally manufactured opioid use. Among the 23 patients, 20 (87%) underwent comprehensive urine drug screening (liquid chromatography-quadrupole time of flight-mass spectrometry testing) during their hospitalization. Medetomidine parent compound was detected in two patients; the test was technically unable to detect medetomidine metabolites. As concern grew about an emerging outbreak of a novel withdrawal syndrome, retrospective analysis of this comprehensive drug screening data was performed for 10 patients after they had been discharged; medetomidine metabolites were detected in all 10 samples* (*5*). These 10 patients with detectable medetomidine metabolites, which included the two with detectable parent medetomidine, were included in the current analysis. A retrospective analysis using a University of Pittsburgh institutional review board–approved single-center toxicology registry was performed.^†^ This approval allows collection and publication of deidentified registry data. 

Of the 10 patients, two arrived at an emergency department (ED) with nausea, vomiting, tremulousness, and autonomic hyperactivity; the other eight experienced similar symptoms several hours after arrival at an ED. All required hospitalization. Nine patients who were admitted to an intensive care unit (ICU) received dexmedetomidine to treat autonomic hyperactivity, and the tenth patient was treated with oral and transdermal clonidine and guanfacine in addition to phenobarbital with sufficient relief to avoid ICU admission (Table). The rationale for using dexmedetomidine included minimal response to other agents including opioids and sedatives, knowledge that medetomidine was an emerging adulterant in the illegal drug supply, and suspicion that medetomidine’s withdrawal state would be effectively treated with dexmedetomidine given the agents’ shared pharmacologic properties. Four patients received treatment for concomitant withdrawal syndromes (e.g., from alcohol or benzodiazepine). None had alternative causes identified to better explain their symptoms. The following two cases are representative of the entire 23-patient cohort and are part of the 10 patients with confirmed medetomidine exposure:

**TABLE:** Clinical characteristics of 10 patients who used illegally manufactured opioids and were treated for medetomidine withdrawal — Pittsburgh, Pennsylvania, October 2024–March 2025

Characteristic	No. (%)
**Mean age (range)**	**34.2 (22–54)**
**Male sex**	**6 (60)**
**Race and ethnicity**	
Black or African American, Hispanic or Latino	1 (10)
Black or African American, non-Hispanic	1 (10)
White, non-Hispanic	8 (80)
Rapid clinical deterioration during ED stay*	**8 (80)**
**Concomitant GABAergic withdrawal requiring treatment**	**4 (40)**
**ICU admission**	**9 (90)**
**Total hospital length of stay, hrs (median)^†^**	**93.3**
**Intubation/Mechanical ventilation**	**1 (10)**
**Vital signs **	
Peak temperature (°C), median (range)	37.6 (36.9–38.6)
Peak heart rate (beats per minute), median (range)	165 (133–178)
Peak systolic blood pressure (mm Hg), median (range)	200 (146–236)
Peak diastolic blood pressure (mm Hg), median (range)	114 (91–140)
**Alpha-2 agonist therapy**	
Dexmedetomidine infusion	9 (90)
Clonidine (cumulative dose),^§^ mg, median	0.7
Tizanidine (cumulative dose),^§^ mg, median	8
Guanfacine (cumulative dose),^§^ mg, median	3
**Cumulative benzodiazepine dose,^§,¶^ mg, median**	**10**
**Cumulative phenobarbital dose,^§^ mg, median**	**520**
**Clinical outcome**	
Cardiac arrest or ventricular dysrhythmia	1 (10)
Metabolic acidosis	7 (70)
Acute kidney injury	3 (30)
Rhabdomyolysis	1 (10)
Myocardial injury	3 (30)
Compounds detected in urine**	
Fentanyl parent, metabolite, or analog	10 (100)
Xylazine	6 (60)
Cocaine parent or metabolite	4 (40)
Lorazepam, temazepam, or diazepam metabolite	6 (60)
Medetomidine parent^††^	2 (20)
Medetomidine metabolite^††^	10 (100)

**Abbreviations: **ED = emergency department; ICU = intensive care unit.

* Patient’s condition in an ED initially assessed as mild, but quickly deteriorated requiring rapidly escalating interventions and an ICU.

^†^ Total hospital length of stay = time (in hours) from initial ED presentation to discharge.

^§^ Cumulative dose is the sum of doses within the first 48 hours of hospital stay beginning from arrival at an ED.

^¶^ Reported in lorazepam equivalents: 1 mg lorazepam = approximately 2 mg midazolam = approximately 5 mg diazepam.

** Urine drug testing conducted using liquid chromatography quadrupole time of flight mass spectrometry.

^††^ One patient had received pharmaceutical dexmedetomidine before collection of urine drug sample.

**Patient A** was a man aged 39 years with opioid use disorder treated with daily methadone. He was seen in an ED for nausea and vomiting, and he had bradycardia (heart rate = 54 beats per minute [bpm]) in triage. Over the next 5 hours, he developed tremulousness, agitation, and severe autonomic hyperactivity: tachycardia (103–170 bpm) and diaphoresis. Treatment with methadone, clonidine, tizanidine, benzodiazepines, and phenobarbital did not result in substantial improvement. Tachycardia improved within 3 hours after dexmedetomidine initiation and admission to an ICU. He was discharged on hospital day 5. Results of enzyme immunoassay urine drug screening collected in an ED after treatment initiation were positive for fentanyl, barbiturates, and benzodiazepines. Comprehensive urine drug screening, collected in the ED with results available on day 3, demonstrated fentanyl and fentanyl analogs, methadone, and medetomidine.

**Patient B** was a man aged 36 years with opioid use disorder who sought treatment in an ED for opioid withdrawal. During 3 hours in the ED, he developed progressive tachycardia up to 163 bpm, hypertension up to 156/134 mmHg, agitation, encephalopathy, severe metabolic acidosis (venous blood pH <6.8), hypokalemia (2.5 mmol/L [reference value = 3.4-5 mmol/L]), and prolongation of the corrected QT interval (QTc = 526 ms). He underwent rapid sequence endotracheal intubation using rocuronium and etomidate, which was complicated by cardiac arrest requiring defibrillation, cardiopulmonary resuscitation, and epinephrine. He was admitted to an ICU and received multiple sedating infusions, including dexmedetomidine, ketamine, propofol, midazolam, and fentanyl. He was extubated several days later without apparent neurologic deficits. Comprehensive urine drug screening, collected in the ED and available on hospital day 3, detected fentanyl and fentanyl analogs, cocaine, ketamine, and sulfamethoxazole-trimethoprim but no parent medetomidine was detected. He was discharged home on hospital day 8. Post-discharge analysis of his urine drug specimen detected medetomidine metabolites. 

## Preliminary Conclusions and Actions

The 10 patients who used illegally manufactured opioids with confirmed medetomidine exposure based on retrospective identification of medetomidine metabolites exhibited a withdrawal syndrome characterized by severe autonomic hyperactivity with rapid symptom onset often requiring dexmedetomidine and ICU admission. The emergence of this syndrome temporally correlated with an increased medetomidine prevalence in the regional illegally manufacture opioid supply. On December 18, 2024, the Pennsylvania Department of Health issued a health advisory describing severe withdrawal observed in patients who use drugs in the Philadelphia area associated with medetomidine exposure.^§^ Although only two patients had detectable parent medetomidine on comprehensive urine drug screening, all 10 patients had samples with detectable medetomidine metabolites identified retrospectively. Although a rapid clinical test for medetomidine is not available, providers should maintain awareness of this emerging medetomidine withdrawal syndrome when treating persons who use illegally manufactured opioids.

## Footnotes

* Mass spectrometry datasets were accessible for retrospective analysis for medetomidine metabolites.

† All chart review was done by two authors who are emergency physicians and medical toxicology fellows using a standardized data abstraction form in REDCap. Source information included toxicology and intensive care unit clinical notes, vital signs, laboratory data, and medication administration records.

§
Hospitals and Behavioral Health Providers are Reporting Severe and Worsening Presentations of Withdrawal among People who Use Drugs (PWUD) in Philadelphia
